# Commentary on the clinical study of CBD-rich cannabis extract in children with autism spectrum disorder

**DOI:** 10.47626/2237-6089-2024-1010

**Published:** 2025-02-27

**Authors:** Claudio Córdova, Otavio Nóbrega

**Affiliations:** 1 Universidade de Brasília Brasília DF Brazil Universidade de Brasília, Brasília, DF, Brazil.

The article “Evaluation of the efficacy and safety of cannabidiol-rich cannabis extract in children with autism spectrum disorder: randomized, double-blind, and placebo-controlled clinical trial”^1^ is a clinical study that evaluated the efficacy and safety of cannabis extract rich in cannabidiol (CBD) in children with autism spectrum disorder (ASD). The heterogeneity of ASD cases was among the positive points of the study, which reflects the clinical reality of patients with frequent comorbidities. The balance between inclusion/exclusion criteria increases the sample representativeness, providing a comprehensive and realistic view of the efficacy and safety of interventions.

However, the authors interpreted important results relying on statistical significance tests and p-values for inferences. A p-value which exceeds an arbitrary level of statistical significance (α) does not guarantee that the effect size (ES) produced by the intervention has practical implications or clinical relevance.^2^ Thus, the clinical impact of the variables under investigation, meaning the clinical significance of the main findings, was lost in the discussion. This lack of focus on clinical significance prevents adequate assessment of the real impact of CBD treatment on the quality of life of family members and children diagnosed with ASD. A more detailed analysis of ES is essential to provide understanding of the treatment effectiveness.^3,4^ This is the aspect of greatest interest to clinicians and readers of this prestigious journal: not only understanding whether the results are statistically significant, but also their relevance and clinical applicability.

The absence of interval estimates represents a significant omission. Observed effects (point estimates) should not be presented in isolation due to the sampling variability inherent in the investigation of any random phenomenon. Confidence intervals (CI) provide a precision measure of the 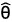 sample estimates for the true θ value. For example, although the authors did not observe a significant difference for the aggressiveness variable (P = 0.2149), the data suggest a favorable effect to the treated group of 0.58. Also for this variable, the 95%CI that I calculated from the study data [-0.05 to 1.21] suggests a range of compatible values for the θ value, meaning from effects without clinical importance [-0.05] to a significant difference of 1.21 (~129% above the observed effect) favorable to the treatment group in reducing aggressiveness. Therefore, although this result is not positive (P > α), it suggests that CBD may have promising effects on this important variable. In order to provide insights to better interpret this result, the *post hoc* statistical power was estimated and proved to be insufficient (1 – β ≈ 44%), possibly not due to the observed ES, but due to the small sample size.

In summary, it is essential to present sufficient information that answers the questions that motivated the study in order to more accurately assess the value of a new therapeutic proposal. Conclusions about the potential benefits of a new therapy cannot be adequately reached without considering the ES of the treatment and the degree of statistical evidence.
